# *Ocimum basilicum* L. Methanol Extract Enhances Mitochondrial Efficiency and Decreases Adipokine Levels in Maturing Adipocytes Which Regulate Macrophage Systemic Inflammation

**DOI:** 10.3390/molecules27041388

**Published:** 2022-02-18

**Authors:** Pandurangan Subash-Babu, Hussah Mohammed Alowaidh, Laila Naif Al-Harbi, Ghalia Shamlan, Amal A. Aloud, Sahar Abdulaziz AlSedairy, Ali Abdullah Alshatwi

**Affiliations:** Adipogenesis and Immunobiology Research Laboratory, Department of Food Sciences and Nutrition, College of Food and Agriculture Sciences, King Saud University, P.O. Box 2460, Riyadh 11451, Saudi Arabia; sbpandurangan@ksu.edu.sa (P.S.-B.); 435203026@student.ksu.edu.sa (H.M.A.); lalharbi1@ksu.edu.sa (L.N.A.-H.); shamlana@ksu.edu.sa (G.S.); aaloud@ksu.edu.sa (A.A.A.); ssudairy@ksu.edu.sa (S.A.A.)

**Keywords:** basil seed, adipocytes, mitochondrial thermogenesis, lipogenesis, inflammation

## Abstract

Excessive storage of lipids in visceral or ectopic sites stimulates adipokine production, which attracts macrophages. This process determines the pro- and anti-inflammatory response regulation in adipose tissue during obesity-associated systemic inflammation. The present study aimed to identify the composition of *Ocimum basilicum* L. (basil) seed extract and to determine its bio-efficacy on adipocyte thermogenesis or fatty acid oxidation and inhibition of lipid accumulation and adipokine secretion. *Ocimum basilicum* L. seed methanol extract (BSME) was utilized to analyze the cytotoxicity vs. control; lipid accumulation assay (oil red O and Nile red staining), adipogenesis and mitochondrial-thermogenesis-related gene expression vs. vehicle control were analyzed by PCR assay. In addition, vehicle control and BSME-treated adipocytes condition media were collected and treated with lipopolysaccharide (LPS)-induced macrophage to identify the macrophage polarization. The results shown that the active components present in BSME did not produce significant cytotoxicity in preadipocytes or macrophages in the MTT assay. Furthermore, oil red O and Nile red staining assay confirmed that 80 and 160 μg/dL concentrations of BSME effectively arrested lipid accumulation and inhibited adipocyte maturation, when compared with tea polyphenols. Gene expression level of adipocyte hyperplasia (*CEBPα*, *PPARγ*) and lipogenesis (*LPL*)-related genes have been significantly (*p* ≤ 0.05) downregulated, and mitochondrial-thermogenesis-associated genes (*PPARγc1α*, *UCP-1*, *prdm16*) have been significantly (*p* ≤ 0.001) upregulated. The BSME-treated, maturing, adipocyte-secreted proteins were detected with a decreased protein level of leptin, TNF-α, IL-6 and STAT-6, which are associated with insulin resistance and macrophage recruitment. The “LPS-stimulated macrophage” treated with “BSME-treated adipocytes condition media”, shown with significant (*p* ≤ 0.001) decrease in metabolic-inflammation-related proteins—such as PGE-2, MCP-1, TNF-α and NF-κB—were majorly associated with the development of foam cell formation and progression of atherosclerotic lesion. The present findings concluded that the availability of active principles in basil seed effectively inhibit adipocyte hypertrophy, macrophage polarization, and the inflammation associated with insulin resistance and thrombosis development. *Ocimum basilicum* L. seed may be useful as a dietary supplement to enhance fatty acid oxidation, which aids in overcoming metabolic complications.

## 1. Introduction

Imbalance between energy intake and followed energy expenditure results in excessive accumulation of triglyceride in circulation [1,2]. Circulating triglycerides consist of three molecules of fatty acid esters with glycerol, which amalgamate in adipocyte’s cytosol and form as lipid droplets, resulting in fat cells [3]. This specialized fat storage in adipocyte is regulated by adipogenic transcription factors, such as adiponectin, peroxisome proliferator activated receptor-γ (*PPARγ*), cytosine–cytosine–adenosine–adenosine–thymidine (CCAAT)–enhancer-binding proteins (*C/EBP*) and sterol-regulatory-element-binding protein-1c (*SREBP-1c*) [2,4]. Adipogenesis have been majorly regulating by *adiponectin* and *PPARγ* expression [4], in addition to chemerin (adipocyte-secreted protein), the angiotensin system and estrogen receptors [5]. Less adipocyte differentiation and excessive adipocyte lipid accumulation are quite similar, which increases the risk of insulin resistance and systemic inflammation associated with metabolic disorders [6]. Insulin sensitivity in hypertrophic adipocytes have been regulated by MMP3 (matrix metalloproteinase-3), which controls adipocyte size and insulin resistance [7].

During obesity progression, the subcutaneous adipose tissue (SAT) decrease, which enhances the storage of excessive lipids in both visceral depots as well as in the ectopic sites [3]. Chronic storage of excessive lipids in visceral or ectopic sites is associated with the production of adipokine(s), involved in energy homeostasis, metabolic complications and inflammation [8]. The predominant and classical adipokines associated with systemic inflammation have been found in pre-condition and mature adipocyte’s stromal vascular fraction (SVF) and in adjacent cells associated with adipose tissue [9]. Adipokines determine the regulation of the pro- and anti-inflammatory response in adipose tissue during the development of obesity and in response to infection or systemic inflammation [10].

In normal conditions, mitochondria play an important role, primarily in fatty acid oxidation (FAO) and the production of adenosine triphosphate (ATP) [11]. The substrate, long chain fatty acyl CoA have been catalyzed by mitochondrial carnitine palmityl transferase 1 (CPT1) enzyme, which is the key step, which ends with CO_2_ production or ketone bodies production to provide energy [12]. In obesity conditions, high TG levels stimulate many inflammatory markers, such as tumor necrosis factor-*α* (TNF-*α*) and reactive oxygen species (ROS), which induce mitochondrial damage and decrease mitochondrial membrane potential (Ψm) [13]. Furthermore, it activates the redox-sensitive inflammatory factor, the nuclear factor kappa-light-chain-enhancer of activated B cells (*Nf-Kb*) [14]. This condition attracts the macrophages into oxidative stressed adipocytes, resulting in inflammatory cytokine development and ending in chronic inflammatory disorders, such as decreased insulin signaling. Furthermore, diminished insulin signaling leads to type 2 diabetes, atherosclerosis and associated inflammatory conditions [15].

*Ocimum basilicum* L. (Basil) seeds have been used in traditional medicine and are consumed as a spice and for flavor in the food industry worldwide. The essential minerals, amino acids and phytochemicals (such as orientine, vicentine and rosmarinic acid) present in *O. basilicum* have been reported for their antioxidant, anti-inflammatory [16] and in silico anti-obesity actions [17], among many beneficial activities [18]. In the present study, we selected *O. basilicum* seeds to analyze the lipid lowering effect and adipokine levels in maturing adipocyte. The bio-efficacy of *O. basilicum* methanol extract has been determined using cytotoxicity analysis. Furthermore, the anti-obesity effect has been evaluated upon lipid accumulation inhibition potential and mitochondrial thermogenesis analysis. In addition, *O. basilicum*-extract-treated adipocytes’ stromal vascular fractions were treated with macrophages to determine macrophage polarization and progression of obesity-associated systemic inflammation. The expected findings allow exploration of the potential of basil seed on adipocyte mitochondrial fatty acid oxidation, lipolysis and macrophage immunoregulation during adipogenesis.

## 2. Materials and Method

### 2.1. Chemicals and Cell Culture

Human mesenchymal stem cells (hMSCs) (American Type Culture Collection (ATCC)) were cultured in Dulbecco’s Modified Eagle’s medium (DMEM) (Sigma, St. Louis, MO, USA) under 5% CO_2_ in 37 °C. Cell growth media have been prepared with 10% fetal bovine serum (Gibco, Paisley, UK) with 1% penicillin/streptomycin (Thermo Fisher, Waltham, MA, USA). Tea polyphenols, rosiglitazone, human insulin, 1-methyl, 3-isobutyl xanthine (IBMX), dexamethasone and all the other molecular-biology-grade chemicals used in this study were purchased from Sigma chemicals (St. Louis, MO, USA).

#### Plant Material

*Ocimum basilicum* L. (Basil seed) were purchased from local market, Riyadh, Saudi Arabia. The *Ocimum basilicum* L. seed were identified and characterized by Dr. V. Duraipandiyan, Herbarium, Department of Botany and Microbiology, College of Science, King Saud University, Riyadh. The voucher specimen, KSU-OB-04 for *O. basilicum* L. have been deposited and maintained in the Public Herbarium, College of Science, King Saud University, Riyadh, Saudi Arabia.

### 2.2. Preparation of Basil Seed Hexane (Non-Polar) and Methanol (Polar) Extract

Basil seed were sieved, milled and ground using a commercial blender. Initially, 200 gm of basil seed powder was soaked in 600 mL of n-hexane for 72 h with frequent shaking using a shaker. After extraction with hexane, the fat content of basil seed was filtered, separated and stored at −20 °C until further use. The residue was again soaked with 600 mL of methanol for 72 h, as sequential extraction. The methanolic extracts of the basil seed, containing fat free phytochemicals, were filtered and stored at −20 °C until further use.

### 2.3. Gas Chromatography and Mass Spectroscopy Analysis

The determination of phytochemicals in hexane and methanolic extract of basil seed was carried out by gas chromatography/mass spectroscopy (GC/MS) analysis. Briefly, GC-MS analysis were performed on a PerkinElmer Clarus 600 GC System, fitted with a Rtx-5MS capillary column (30 m × 0.25 mm inner diameter, ×0.25 μm film thickness; maximum temperature, 350 °C), coupled to a Perkin Elmer Clarus 600C MS. Ultra-high purity helium (99.99%) was used as carrier gas at a constant flow rate of 1.0 mL/min. The injection, transfer line and ion source temperatures were all 290 °C. The ionizing energy was 70 eV. Electron multiplier voltage was obtained from autotune. The oven temperature was programmed from 60 °C (hold for 2 min) to 280 °C at a rate of 3 °C/min. The crude samples were diluted with appropriate solvent (1/100, *v*/*v*) and filtered. The particle-free diluted basil seeds extracts (1 μL) were taken in a syringe and injected into injector with a split ratio 30:1. All data were obtained by collecting the full-scan mass spectra within the scan range 40-550 AMU. The percentage compositions of the basil seeds extract constituents were expressed as a percentage by peak area (PerkinElmer Clarus, 600 GC System).

### 2.4. Isolation of Monocytes from Whole Blood and Bacterial LPS-Stimulated Macrophage Formation

About 6 mL of human peripheral blood was collected from healthy donors by the qualified medical lab technology professional. Blood cells present in buffy coat were mixed with an equal volume of PBS, layered on Histopaque-1077 (Sigma, St. Louis, MO, USA) and centrifuged at 400× *g* for 30 min at 20 °C. The interface containing mononuclear cells were collected and cultured with DMEM (AG-Biochrom, Berlin, Germany) containing 10% fetal bovine serum (Hi-clone, Logan, UT, USA) in a CO_2_ incubator at 37 °C with 5% CO_2_ for 12 h. After 12 hr incubation, the attached monocytes were utilized for the present study with a density of 10,000 cells/cm^2^ onto 6-well cell culture plate. Monocytes were treated with 10 ng/mL of LPS for 24 h to induce to macrophage polarization and kept in 5% CO_2_ incubator. After 24 h, differentiated macrophages were thoroughly washed 5 times with DMEM containing 10% FBS and the polarized macrophages were utilized for experiments immediately.

### 2.5. Differentiation of Human Mesenchymal Stem Cells into Preadipocytes

Human mesenchymal stem cells (hMSCs) were cultured on self-assembled monolayer at a density of 10,000 cells cm^−2^ were utilized for adipogenic differentiation. Adipocyte differentiation induction medium were freshly prepared with DMEM (10% FBS) contain 1 µM of dexamethasone, 0.5 µM of 3-isobutyl-1-methyl-xanthine (IBMX), 167 nM recombinant human insulin and indomethacin. Differentiation media were replaced to hMSCs and maintained for 72 h in CO_2_ incubator at 37 °C with 5% CO_2_. Immediately after 72 h, cells were replaced with maintenance media containing recombinant human insulin for 48 h [19]. Differentiated preadipocytes were confirmed morphologically under inverted microscope and immediately utilized for the experiment.

### 2.6. Cytotoxicity Assay

The cytotoxicity of basil seed methanol extract (BSME) was determined using a modified MTT assay [20]. Briefly, cells were seeded at a density of 5000 cells/well onto a flat-bottomed 96-well culture plate and were treated with increasing concentration (0, 10, 20, 40, 80, 160 and 320 μg/dL, dissolved in DMSO) of BSME extracts for 24 h and 48 h, respectively. Cells were labeled with MTT solution (1 mg/mL in phosphate-buffered saline [PBS]) for 4 h and the resulting formazan were solubilized in 10% DMSO. The absorption was measured at λ = 570 nm using multi-well plate reader.

### 2.7. Experimental Design

Preadipocytes were treated with increasing concentrations of BSME for 14 days, to determine the effect on adipocyte’s fatty acid metabolism regulation. Initially (on day 0), vehicle control, 40 μg/dL, 80 μg/dL and 160 μg/dL doses of BSME were treated to preadipocytes. On day 3, BSME-treated preadipocytes were replaced with maintenance medium containing 40 μg/dL, 80 μg/dl and 160 μg/dL doses of BSME, respectively, and maintained until day 6. However, vehicle control was replaced with maintenance medium. In continuation from day 7 to day 14, in all the groups, the media was replaced with maintenance medium once every 3 days. In addition, a reference drug, tea polyphenol (160 μg/dL), was also treated with the same experimental design. On day 14, the experimental cells’ condition media (containing BSME- or tea-polyphenol-treated, adipocyte-secreted and cellular proteins) have been collected and the adherent cells were processed for lipid accumulation and gene expression analysis accordingly.

BSME- and tea-polyphenol-treated adipocyte condition media and normal growth media (1:1 ratio) were supplemented to LPS-stimulated macrophage and maintained for 12 h. Vehicle control and negative control groups were treated with DMSO alone. At the end of experiment, the condition media was collected for inflammatory cytokine quantification. Total RNA was extracted from experimental cells to quantify relative gene expression.

### 2.8. Light (Oil Red O) and Fluorescent (Nile Red) Staining Analysis for Lipid Accumulation

Oil red O and Nile red staining analysis were determined using modified method of Park et al. [21]. Briefly, 500 mg of oil red O in 100 mL of 100% isopropanol was prepared as stock solution and the working solution was prepared with 3:2 ratio of stock with 60% isopropanol. A measure of 200 μL of working oil red O solution was added to vehicle control and BSME-treated (40 μg/dL, 80 μg/dL and 160 μg/dL) maturing adipocytes after being fixed with 4% formaldehyde. After 60 min incubation in room temperature, the unbound oil red O were removed by PBS washing and images were immediately analyzed using an inverted light microscope. The accumulated oil red O from the processed cell were allowed to dry overnight, then the stains were extracted and quantified after dissolving with isopropanol and absorbance was measured at 520 nm.

Fluorescent Nile red staining was performed to determine the lipid accumulation using fluorescence microscopy. Briefly, 4% formaldehyde-fixed vehicle control and BSME-treated experimental cells were stained with Nile red fluorescence (5 mg in 1 mL of 100% acetone) for 30 mints at 37 °C. After incubation, the accumulations of fluorescence were immediately captured with an inverted fluorescence microscope.

### 2.9. Mitochondrial Membrane Potential (JC-1 Staining) Assay

Mitochondrial membrane potential was determined using JC-1 dye to assess mitochondrial efficiency in vehicle control, 40 μg/dL, 80 μg/dL and 160 μg/dL doses of BSME-treated adipocytes. Briefly, JC-1 staining solution (mixed with equal volume of culture medium) was added to adipocyte and incubated for 20 min in the dark at 37 °C. After incubation, the unbound JC-1 dye have been gently removed by washing with 200 μL of JC-1 staining wash buffer at 4 °C and this process was repeated 2 times. Then, the fluorescence was observed using fluorescence microscope and images were captured.

### 2.10. Biochemical Parameters Analysis

The amount of triglyceride (TG) and free glycerol level in the vehicle control and the BSME-treated (80 and 160 μg/dL) adipocytes were quantified by commercial kit method (Abcam, Austria) [22]. Activity of lactate dehydrogenase (LDH) was measured using enzymatic assay kit (Abcam, Austria). The protein contents were quantified by the Bradford method [23].

### 2.11. Quantitative Polymerase Chain Reaction (qPCR) Analysis

The vehicle control and the BSME-treated (80 and 160 μg/dL) cells (adipocyte and macrophage) total RNA and cDNA were synthesized using Fastlane^®^ Cell cDNA kit using qPCR. Adipocyte hyperplasia (*C/EBPα*, *PPARγ*, *HSL* and *LPL*), fatty acid oxidation and energy expenditure (*adiponectin-R1*, *PPARγC1α*, *UCP-1*, *PRDM16*, *SREBP-1c* and *FABP-4*) in adipocytes. The insulin resistance and metabolic inflammation (*IL1β*, *IL12β1*, *IL-6*, *IL-4*, *TLR-4*, *IL-33*, *IKBKγ1*, *TNFα*, *NF-κB* and *TGFBR2*)-related genes and the reference gene, β-actin, were analyzed in the macrophage, according to the method of Yuan et al. [24]. The amplification values (ΔCt) have been calculated by the difference between Ct (treated) and Ct (control). Gene expression were plot using the expression of 2^−ΔΔCt^ value.

### 2.12. Quantification of Protein Using ELISA

The intracellular (stromal vascular fraction) have been prepared using commercially available protein extraction kit (Mammalian protein extraction reagent, Thermo Scientific, Waltham, MA, USA). According to the kit protocol, the vehicle control and the BSME-treated adipocytes’ cell membranes were digested and the whole proteins were collected in respective micro centrifuge tubes. The amount of metabolic inflammation, insulin resistance and fatty acid metabolism deregulating markers, such as leptin, *TNF-α*, *IL-6* and *STAT-6*, (in adipocytes) and *PGE-2*, *TNF-α*, *MCP-1* and *NF-κB* (in macrophage) were analyzed in vehicle control and BSME-treated cells using high-sensitivity ELISA kits (Quantikine, R&D Systems, Minneapolis, MN, USA). This assay does not distinguish between soluble and receptor-bound proteins and thus gives a measure of the total concentration of inflammatory mediator proteins. The values were expressed as pg/mg protein for all the analyzed proteins.

### 2.13. Statistical Analysis

All the grouped data were statistically evaluated using SPSS/28.5 software package. The values were analyzed by one-way analysis of variance (ANOVA) followed by Tukey’s range test. All the results were expressed as mean ± SD for six replications in each group. *p* values < 0.05 were considered significant [25].

## 3. Results

### 3.1. Identification of Phytochemicals by Gas Chromatography–Mass Spectrometry

**Figure 1:**
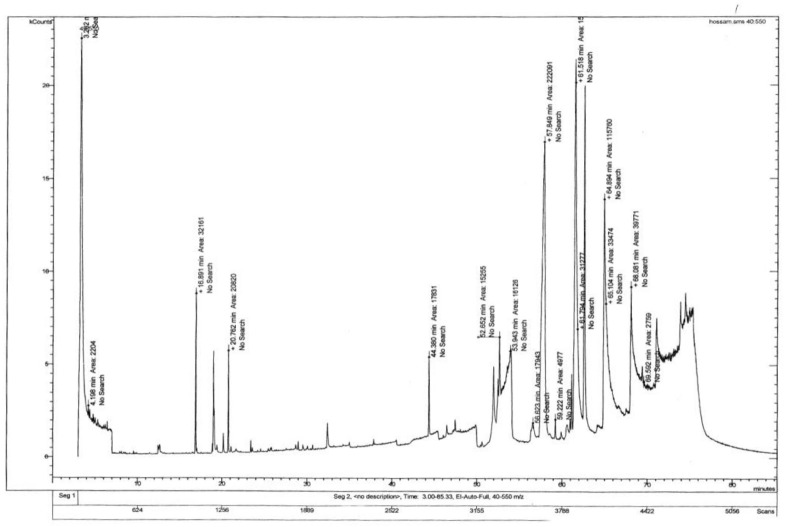
GC-MS spectral data for basil seed methanol extract (BSME).

GC-MS chromatogram of BSME have been presented in Figure 1. Spectral data were compared with NIST-11 library, and the identified phytochemicals and their pharmacological effects are presented in Table 1. We found 95%–99% similarity as per the peak values and retention time, such as ricinoleic acid, gamabufotalin, colchicine, beclomethasone, prednisone, β carotene, levodopa, retinol, triaziquone, retinyl acetate and vincamine. Some of them are known for their biological activity [26], whereas a few compounds remain unexplored (Table 1). In addition, basil seed hexane extract also showed the availability of dihydroxybutanedioic acid, octodriene, colchicine, gamabufotalin, retinol, β carotene and retinyl acetate (spectral data available in Supplementary Materials Figure S1 and Table S1).

### 3.2. Effect of Basil Seed Methanol Extract on Adipocyte and Macrophage Viability

**Figure 2:**
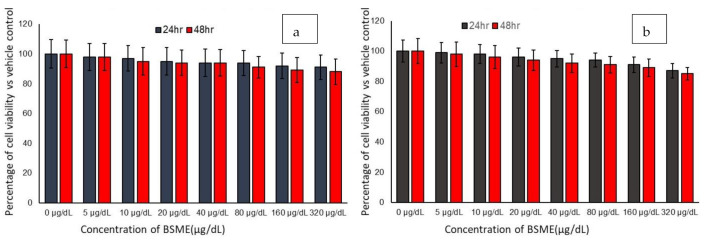
In vitro cytotoxic effect of basil seed methanol extract (BSME) in preadipocytes after 48 h (**a**) and macrophages after 12 h (**b**). Results are presented as the mean ± standard deviation (SD) (*n* = 6 in all the groups).

The higher concentrations of BSME which we tested produced very low percentages of cytotoxicity in both adipocyte and macrophage. In adipocyte, 320 µg/dL of BSME shown 9% reduction in cell viability in 24 h and 12% in 48 h, as presented in Figure 2a. In macrophage, 15% of cell growth inhibition was observed only in 48 h with 320 µg/dL dose of BSME (Figure 2b). According to the present result, BSME have not produced IC_25_ or IC_50_ against preadipocytes or macrophages.

### 3.3. Dose Determination Based on Lipid Accumulation Inhibitory Potential Using Oil Red’ O Staining Analysis

**Figure 3:**
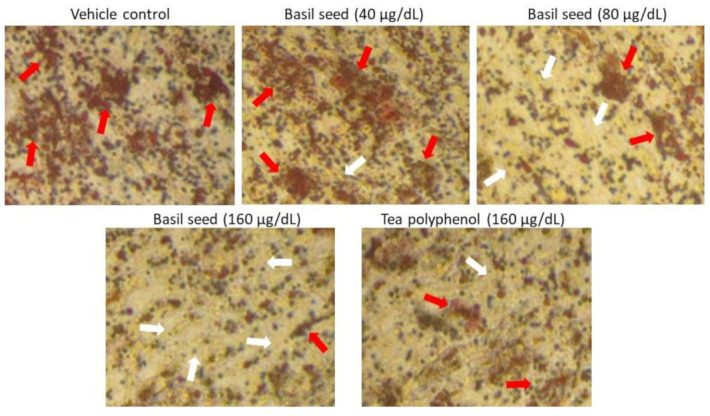
Determination of lipid accumulation using oil red O staining analysis after basil seed methanol extract (BSME) treatment for 14 days. In oil red O staining, vehicle control showing hypertrophic adipocyte (red arrow) was directly propositional to triglyceride storage. However, in 160 µg/dL of BSME treatment, it showed controlled adipocyte maturation, less lipid accumulation and spindle-shaped (white arrow) adipocytes when compared with 40 and 80 µg/dL of BSME-treated and vehicle control cells.

**Figure 4:**
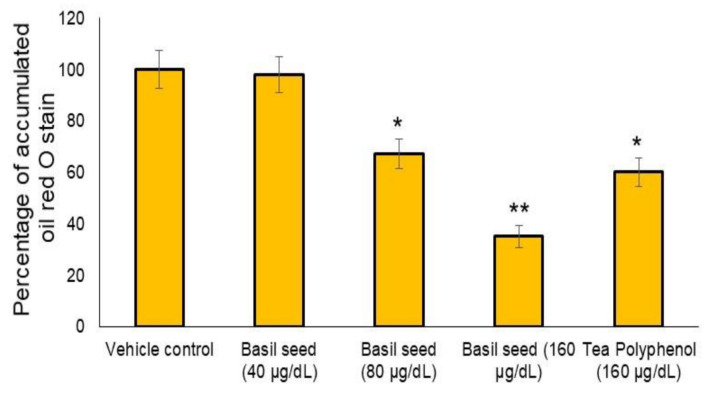
Effective dose determination of basil seed methanol extract (BSME) on inhibitory potential of lipid accumulation after 14 days treatment. Values are means ± SD (*n* = 6). * *p* ≤ 0.05 by comparison with vehicle control. ** *p* ≤ 0.001 by comparison with vehicle control and tea polyphenol.

In the present study, 40, 80 and 160 μg/dL concentration of BSME have been selected to assess lipid accumulation inhibition potential. After 14 days, the results of oil red O staining confirmed that 160 μg/dL of BSME effectively arrested lipid droplets in maturing adipocyte when compared with vehicle control (Figure 3). Results shown that the lipid accumulation was decreased significantly (*p* ≤ 0.001) by 65% in 160 μg/dL and 33% in 80 μg/dL of BSME, when compared with vehicle control (Figure 4). The effect was compared with the reference drug—tea polyphenol—160 μg/dL of tea polyphenol decreased only 40% of lipid accumulation when compared with 160 μg/dL of BSME.

### 3.4. Determination of Lipid Accumulation in Adipocyte Using Nile Red Fluorescence Staining

**Figure 5:**
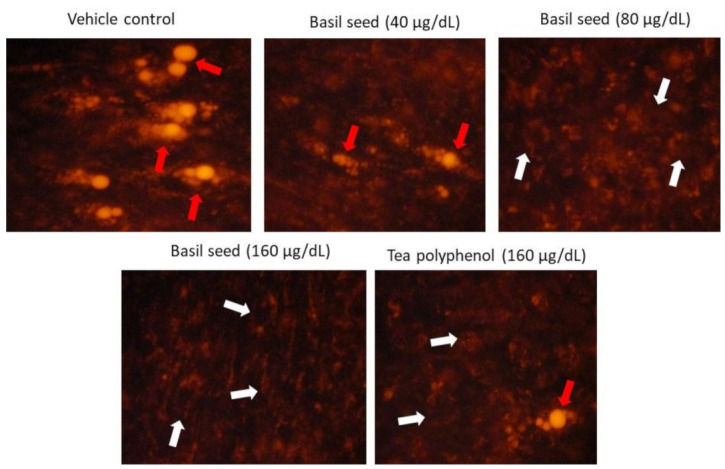
Determination of hypertrophic adipocyte and lipid accumulation using Nile red fluorescent staining analysis after basil seed methanol extract (BSME) treatment for 14 days. In Nile red staining, vehicle control showing high red fluorescence (red arrow), directly propositional to accumulation of lipid droplets. However, 160 µg/dL of BSME treatment showed inhibited adipocyte maturation (white arrow) and less lipid accumulation in adipocyte when compared with lower doses of basil-seed-treated maturing adipocytes.

In vehicle control, Nile red analysis confirmed the hypertrophic and high lipid fluorescence in adipocytes after 14 days. Treatment with BSME significantly (*p* ≤ 0.001) decreased the lipid accumulation and adipocyte hypertrophy in 80 and 160 µg/dL dose was shown in Figure 5. Most interestingly, 160 µg/dL dose of tea polyphenol did not reduce adipocyte hypertrophy, also the effect was lower than the 40 µg/dL dose of BSME (*p* ≤ 0.05).

### 3.5. Activity of Glycerol Phosphate Dehydrogenase, LDH and Level of Triglyceride in Vehicle Control and BSME-Treated Adipocyte after 14 Days

The activity of glycerol phosphate dehydrogenase and lactate dehydrogenase were significantly (*p* ≤ 0.001) decreased in 80 and 160 µg/dL dose of BSME-treated matured adipocyte after 14 days. The observed effect was significantly (*p* ≤ 0.001) higher than the reference drug, tea polyphenol, as presented in Table 2. In addition, a significantly lower level of triglyceride was found in BSME when compared with tea polyphenol (160 µg/dL) or with a lower dose (80 µg/dL) of BSME. Based on the observations of biochemical parameters and lipid accumulation analysis results, we selected the 160 µg/dL dose of BSME for further gene and protein expression analysis.

### 3.6. Mitochondrial Membrane Potential (JC-1) and Oxidative Efficiency Analysis

**Figure 6:**
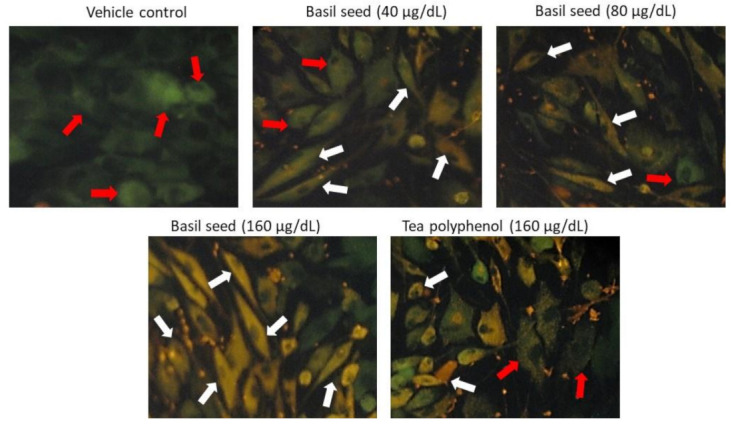
Mitochondrial membrane potential, JC-1 (c) staining images (200×) of vehicle control and basil-seed-methanol-extract-treated (BSME) adipocytes after 14 days. JC-1 fluorescence images showing merged images of the red and green signals of the dye, corresponding to JC-1 in J-aggregates vs. monomeric form. We found less J-aggregates and hypertrophic adipocyte (red arrow) in vehicle control, and in 40 µg/dL and 80 µg/dL of BSME-treated adipocytes. In 160 µg/dL of BSME, showing linear and spindle-shaped adipocytes with high j-aggregates (white arrow), showed high MMP, directly representing active mitochondria.

Mitochondrial membrane potential (MMP) is predicted by the oxidative capacity of fatty acid and energy metabolism. Equal volumes of culture medium and JC-1 staining solution were mixed and added to vehicle control, BSME-treated (160 µg/dL) and tea-polyphenol-treated (160 µg/dL) adipocytes, respectively. In Figure 6, BSME-treated groups showing the images of JC-1 staining clearly represent merged images of dye having red and green signals, corresponding to JC-1 in J-aggregates vs. monomeric form. We found 40 and 80 µg/dL of BSME shown hypertrophic adipocytes with less J-aggregates, confirming less mitochondrial potential. However, 160 µg/dL of BSME showed linear and spindle-shaped adipocytes with high J-aggregates, directly representing the potential of mitochondrial efficiency on thermogenesis.

### 3.7. Quantification of Adipogenesis, Mitochondrial Thermogenesis and Inflammation-Related Gene Expression Levels in BSME-Treated Adipocytes

**Figure 7:**
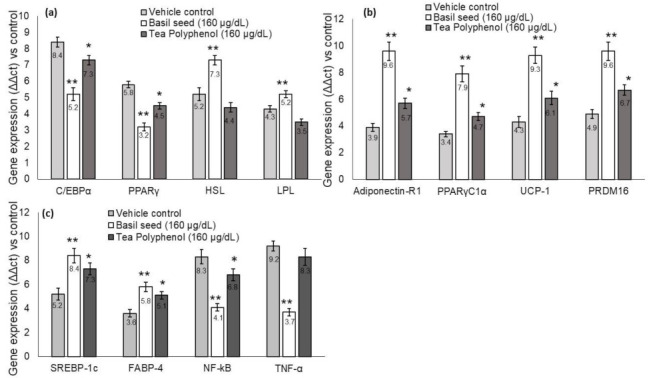
Effect of basil seed methanol extract (BSME) on adipocyte hyperplasia (**a**), lipolysis (**b**) and metabolic-inflammation-related (**c**) gene expression levels after 14 days. Values are means ± SD (*n* = 6). * *p* ≤ 0.05 by comparison with vehicle control and ** *p* ≤ 0.001 by comparison with vehicle control and tea polyphenol.

We found significantly (*p* ≤ 0.001) decreased mRNA expression levels *C/EBPα, PPARγ*, increased lipoprotein lipase (*LPL*) and hormone-sensitive lipase (*HSL*) expression levels in BSME (160 µg/dL), when compared with vehicle control and tea polyphenol. Most interestingly, we observed significantly increased adipocyte mitochondrial-efficiency-related mRNA expression, such as *PPARγC_1_α*, *adiponectin-R1*, *UCP-1*, *SREBP1c*, *FABP4* and *PRDM16*, have been presented in Figure 7. The metabolic-inflammation-related genes, such as *NF-kB* and *TNF-α* expressions, have been decreased in BSME-treated (160 µg/dL) adipocytes. This evidences that basil seed treatment effectively restores the hypertrophic adipocyte and the associated metabolic inflammation in maturing adipocytes.

### 3.8. Intracellular Protein Levels in BSME-Treated Adipocyte after 14 Days

**Figure 8:**
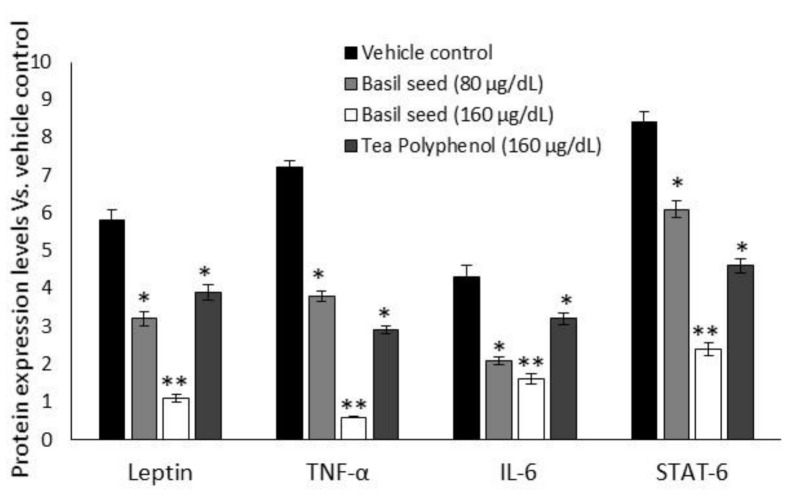
Effect of basil seed methanol extract (BSME) on insulin resistance and metabolic-inflammation-related protein (leptin, TNF-α, IL-4 and STAT-6) levels in maturing adipocytes after 14 days. Values are means ± SD (*n* = 6). * *p* ≤ 0.05 by comparison with vehicle control and ** *p* ≤ 0.001 by comparison with vehicle control and tea polyphenol.

Figure 8 shows the results for adipokines and insulin-resistance-related intracellular protein levels of BSME-treated (160 µg/dL) adipocyte’s stromal vascular fractions. We found significantly (*p* ≤ 0.001) decreased levels of leptin, TNF-α, IL-6 and STAT-6 proteins, when compared with vehicle control or tea polyphenol treatment.

### 3.9. Results for Macrophage Treated with “BSME-Treated Adipocyte Condition Media”

#### 3.9.1. Determination of Lipid Accumulation and Foam Cell Development in Macrophage Using Oil Red O Staining

**Figure 9:**
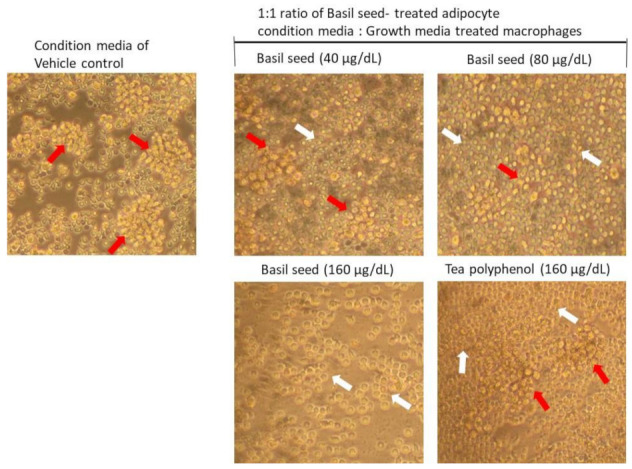
Determination of lipid accumulation and macrophage foam cell in basil-seed-treated adipocyte condition media-treated polarized macrophages using oil red O staining analysis after 12 h. Condition media of vehicle control macrophages showed hyper proliferating cells with clumped morphology representing foam cell development (red arrows). Basil seed 40 µg/dL 80 µg/dL and tea polyphenol (160 µg/dL) dose showed a reduced number of proliferating and clumped cells. In 160 µg/dL dose of basil seed showing minimal number of cells, less proliferation and uniform morphology of macrophages (white arrows).

LPS-stimulated macrophages were treated with BSME-treated adipocyte condition media were analyzed for development of foam cell and atherosclerotic lesion after 12 h. As presented in Figure 9, we observed an increased foam cells or inflated macrophage in adipocyte condition media treated (vehicle control) macrophage. However, 80 or 160 μg/dL dose of BSME-treated adipocyte condition media effectively reduced the inflated macrophage and foam cell percentage (*p* ≤ 0.001).

#### 3.9.2. Metabolic-Inflammation-related Gene Expression Levels in Macrophage after Treatment with BSME-Treated Adipocyte Condition Media

**Figure 10:**
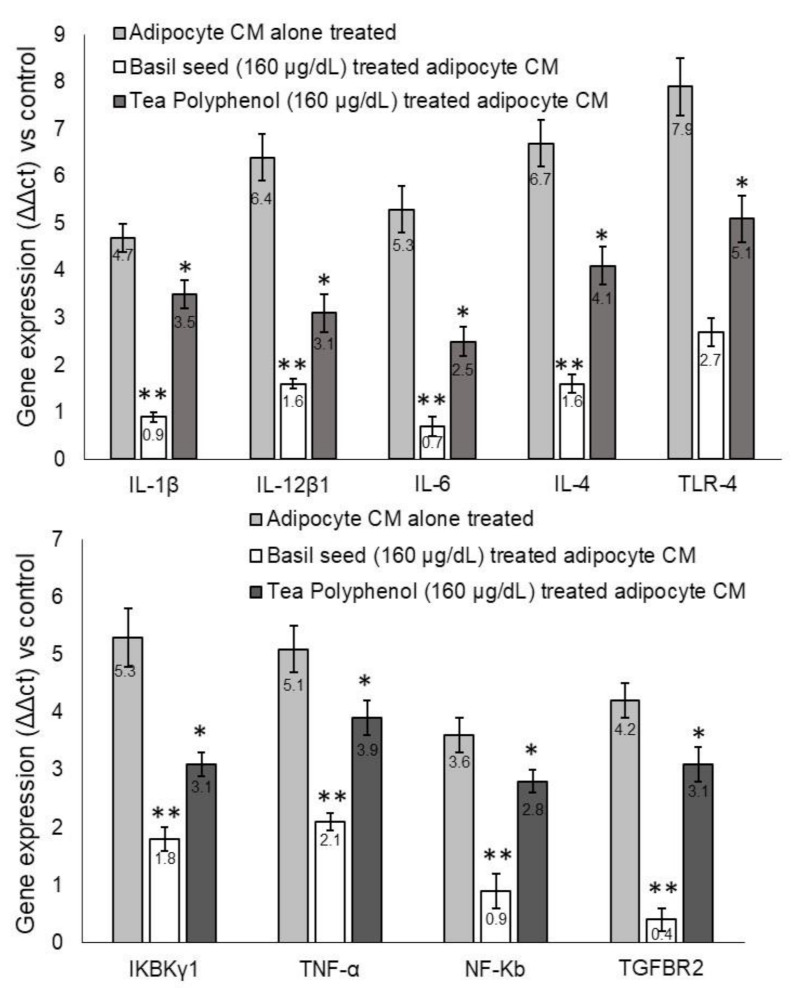
Quantification of metabolic-inflammation-related gene expression levels in basil-seed-methanol-extract-treated (BSME) adipocyte condition media treated macrophage after 12 h. Values are means ± SD (*n* = 6). * *p* ≤ 0.05 by comparison with vehicle control. ** *p* ≤ 0.001 by comparison with vehicle control and tea polyphenol.

Gene expression analysis shown that pro-inflammation related genes *IL-1β*, *IL-12β1*, *IL-6*, *IL-4* and *TLR-4* have been upregulated in untreated adipocytes and condition-media-treated macrophages. In contrast, treatment with BSME-treated adipocyte condition media (1: 1 ratio) for 12 h significantly (*p* ≤ 0.001) downregulated the metabolic-inflammation-related genes *IL-1β*, *IL-12β1*, *IL-6*, *IL-4* and *TLR-4* genes in macrophages. The observed effect was more significant (*p* ≤ 0.001) when compared with tea polyphenol (160 μg/dL) (Figure 9). In addition, we found that the expression levels of *IKBKγ1*, *TNFα*, *NF-κB* and *TGF-βR*_2_ were downregulated in BSME-treated macrophages when compared with vehicle control after 12 h. The observed effect was more significant (*p* ≤ 0.001) than reference drug, tea polyphenol (160 μg/dL) (Figure 10).

#### 3.9.3. Quantification of Intracellular Protein Levels in Macrophage after Treatment with “BSME-Treated Adipocyte Condition Media”

**Figure 11:**
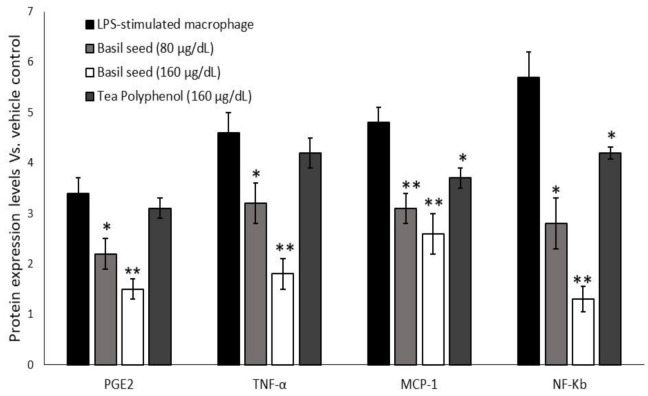
Quantification of intracellular protein levels in macrophages treated with basil-seed-treated adipocyte condition media after 12 h. Values are means ± SD (*n* = 6). * *p* ≤ 0.05 by comparison with vehicle control. ** *p* ≤ 0.001 by comparison with vehicle control and tea polyphenol.

As presented in Figure 11, we found an increased levels of PGE-2, TNF-α, MCP-1 and NF-κB in vehicle control, they majorly associated with macrophage colony stimulation and metabolic vascular inflammation. In contrast, BSME-treated adipocytes and condition-media-treated macrophage did not show higher expression of macrophage colony stimulation factor and PGE-2 and MCP-1 level. In addition, PGE-2 and NF-κB significantly (*p* ≤ 0.001) suppressed in BSME-treated adipocytes and condition-media-stimulated macrophage.

## 4. Discussion

Traditional medicinal plants provide abundant bioactive compounds with proved health-promoting activities, such as anti-obesity, anti-inflammatory and antioxidant actions [18,26]. In the present study, we identified that the major phytochemical compounds from basil seed methanolic extract (BSME) are ricinolic acid, gamabufotalin, colchicine, beclomethasone, prednisone, beta carotene, levodopa, retinol, triaziquone, retinyl acetate and vincamine. The identified compounds, such as ricinoleic acid [27], gamabufotalin [28], colchicine [29], beclomethasone [30] and prednisone [31], have been identified for anti-inflammatory potential. In addition, beta carotene [32], levodopa [33], retinol, retinyl acetate [34], triaziquone [35] and vincamine [36] have been reported for anti-obesity and antioxidant potential. Basil seed has been identified for its potential in antispasmodic and stomachache medicine [37]. Bucktowar et al. [38] discuss the anti-inflammatory, weight loss and anti-cancer activities of basil seeds.

In the present study, we observed that BSME effectively arrested lipid storage in maturing adipocyte via enhancing the mitochondrial fatty acid oxidation, and energy expenditure was confirmed by increased thermogenesis-associated gene expression patterns. In this context, Yang et al. [39] have reported that ethanol extract of black sesame seed has been identified to ameliorate hepatic lipid accumulation, oxidative stress and insulin resistance in fructose-induced nonalcoholic fatty liver disease. BSME effectively increased *adiponectin-R1*, *PPARγC_1_α*, *UCP-1*, *SREBP1c* and *PRDM16* expression, majorly involved in mitochondrial energy metabolism in adipocyte. This effect may be due to the bioactive compounds β carotene and retionol present in BSME, which regulate lipolysis via mitochondria-dependent fatty acid oxidation [32,34]. Enhanced mitochondrial thermogenesis leads to cellular depletion of lipid storage, further suppressing adipocyte-hyperplasia-associated *C/EBPα* and *PPARγ* mRNA levels. Most notably, *HSL* and *LPL* expression levels have been increased in BSME-treated adipocytes, confirming that basil seed effectively increases the metabolism of stored lipids through fatty acid β oxidation. This effect was not observed in vehicle control. Diminished phosphorylation of AMP-activated protein kinase (AMPK) was linked with reduced mitochondrial β oxidation, increased triglyceride storage, oxidative stress—ending with insulin resistance—and inflammation [40]. In response to increased circulatory free fatty acid in the fed state, SREBP-1c activates its lipogenic target genes, such as fatty acid synthase and acetyl CoA carboxylase, which results in increased cellular fatty acid uptake. Further hepatic activation of AMPK, in part through its phosphorylation of SREBP-1c, protected against hepatic steatosis, hyperlipidemia and accelerated atherosclerosis [41]. In the present study, the observed activation of SREBP-1c and FABP-4 levels may be beneficial in significant uptake of circulatory fatty acid ends with reduced hyperlipidemia and metabolic inflammation. In this context, Luo et al. [42] have found a mechanistic anti-obesity effect of flaxseed polysaccharide via inducing satiety by leptin resistance and promoting lipid metabolism through the AMPK signaling pathway. Berndt et al. [43] reported that impaired insulin sensitivity was associated with decreased adipose triglyceride lipase (ATGL) and *HSL* expression, independently of body fat mass and fat distribution.

*PRDM16* and *PPARγC_1_α* are the transcription coregulators, majorly found in densely packed inner mitochondrial membranes, which contain *UCP-1* [19,44]. *UCP-1* initiates the reaction allowing protons to re-enter the mitochondrial matrix without generating ATP. Cellular thermal variation triggers a signal transduction cascade that converts excessive circulating lipids and glucose (nutrients) into acetyl-CoA to generate ATP and heat [45]. The clinical significance of this study reveals that mitochondrial thermogenesis was increased in maturing adipocyte via activation of *adiponectin*, *PPARγC_1_α*, *PRDM16* and *UCP-1* expressions, which arrest adipocyte hypertrophy. In this context, Chani et al. [46] have found that long term intake of chia seed lower lipid deposit in hepatocytes and increased intestinal muscle layer and crypt size in Sprague Dawley rats.

Adipose tissues secrete several adipokines, which exert their biological role in autocrine or paracrine regulation of appetite and energy balance. The adipokines influence several physiological processes, associated with energy homeostasis, glucose metabolism and immunity [47]. Anti-inflammatory cytokines (adiponectin, transforming growth factor-β, IL-4) were produced from the adipose tissue of lean persons with active mitochondria, and they mediated physiological functions instead of metabolic diseases. However, proinflammatory cytokines (TNF-α, IL-6, leptin) were produced by obese adipose tissue modulate insulin signaling via stimulation of inflammatory pathway [48]. TNF-α phosphorylate insulin receptor substrate-1 (IRS-1) has a negative interference with the insulin signaling pathway [49]. Stimulating lipolysis in adipocyte, without inhibiting TNF-α, might be useless, because TNF-α is known to promote lipolysis and inhibit adipocyte maturation, which contributes to higher hepatic glucose production and hyperglycemia. Excessive circulatory glucose engaged to store in visceral adipose tissue via IL-6, STAT-6 and IL-1β have been stimulated by TNF-α [50]. Accumulation of excessive fat in visceral adipose tissue is caused many metabolic-inflammation-related pathophysiological disorders. Many findings confirmed that weight loss reduces plasma TNF-α and IL-6 concentrations, while feeding animals high-fat diets will increase the TNF-α content of their adipose tissue [51]. Stimulation of mitochondrial-dependent fatty acid oxidation regulates peripheral nutrient metabolism by antagonizing the catabolic actions of PPARα in the liver and reducing adipose tissue inflammation. We noticed that the BSME decreased the expression levels of insulin resistance and macrophage chemoattractant proteins, such as TNF-α, IL-6, leptin and STAT-6, in maturing-adipocyte-secreted proteins.

Hypertrophic adipocytes produce inflammatory cytokines (TNF-α, IL-6, IL-1β, and CCL2) which leads to the recruitment of inflammatory cells, such as lymphocytes and macrophages. The immune cell infiltration and accumulation in inflammatory adipose tissue, which establishes a state of low-grade chronic inflammation and obesity-induced inflammation in visceral adipose tissue. Increased levels of CCL2, TNF-α, IL-1, IL-6 and inducible nitric oxide synthase (iNOS) in the visceral adipose tissue of obese people have been very common [52]. We found LPS-stimulated macrophages with increased foam cells or inflated macrophage. However, BSME-treated adipocyte condition media effectively decreased the inflated macrophage and adipose tissue macrophages (foam cell) percentage. Hypertrophic adipocyte-secreted TNF-α, which activates nuclear factor Kb (NF-κB), is a key factor for cell survival and death via proinflammatory signaling cascade [53]. BSME-treated macrophage showed decreased levels of metabolic-inflammation-related mRNA expressions *IL1β*, *IL12β1*, *IL-4*, *IKBKγ1*, *NF-κB*, *TNFα*, *TLR-4* and *TGF-βR2* when compared with the vehicle control. Our findings are in the line with a previous study, showing that the basil extracts reduced the expression of inflammatory cytokine mRNA induced by co-culture, including those of *IL-6*, *IL-1β*, *TNF-α* and *NF-κB* [17].

Hypertrophic adipocyte-secreted TNF-α and monocyte chemoattractant protein-1 (MCP-1), further they attract macrophages and convert adipocyte into adipose tissue macrophages (ATMs) as a mediator of inflammatory responses in adipose tissue [48,54]. These ATMs are the predominant source of proinflammatory cytokines, such as TNF-α and PAI-1, and may be recruited to adipose tissue via MCP-1 [55]. They are mainly associated with the progression of foam cell formation and atherosclerotic lesion development—which further contributes to atherosclerotic plaque instability and thrombus formation. Major metabolic-inflammation-related proteins secreted by macrophages are PGE-2, TNF-α, MCP-1 and NF-ĸB, and are mainly involved in the development and progression of foam cell formation and atherosclerotic lesion—which further contributes to atherosclerotic plaque instability and thrombus formation [48]. BSMS treatment effectively decreased the PGE-2, TNF-α, MCP-1 and NF-ĸB levels in adipocyte condition-media-treated macrophages when compared with vehicle control.

The observed anti-obesity and immunoregulatory mechanistic effect of basil seed might be due to the availability bioactive compounds, such as ricinolic acid, gamabufotalin, colchicine, beclomethasone, prednisone, beta carotene, levodopa, retinol, triaziquone, retinyl acetate and vincamine. Of clinical significance of the present study—the observed lipid lowering effect of basil seed might be due to the increased expression of *PPARγC1α*, *PRDM16*, *UCP-1* and *adiponectin-R1* levels, that stimulate mitochondrial β oxidation and deplete triglyceride storage. Furthermore, metabolically active adipocytes inhibit the origin of adipokine and proinflammatory cytokines associated with macrophage-colony-stimulating factor and foam cell formation.

## 5. Conclusions

Basil seed methanol extract ameliorates adipocyte lipid accumulation and enhances mitochondrial fatty acid oxidation via *PPARγC1α*, *PRDM16* and *UCP-1*. Enhanced adipocyte fatty acid oxidation diminishes adipocyte hyperplasia, via downregulation of *C/EBPα* and *PPARγ* genes, when compared with vehicle control and tea polyphenol. Basil-seed-treated adipocyte stromal fractions showed less monocyte chemoattractant proteins and TNF-α, which decrease the risk of adipose tissue macrophages (ATMs) development as a mediator of inflammatory responses in adipose tissue. The obtained beneficial effect of basil seed on lipid lowering potential may be due to the presence of colchicine, gamabufotalin, beta carotene, vincamine and retinol. Basil seed metabolically activates the adipocyte, which diminishes adipokine and proinflammatory cytokine development, which decrease the progression of insulin resistance and atherosclerosis. So, further in vivo research warrant to confirm the limitations of basil seed to be used as a health supplement or drink to enhance immunity.

## Figures and Tables

**Table 1:** Basil seed methanol extract (BSME) composition analysis using GC–MS showed 99–95% similarity in the database.

Peak No.	List of Compounds	Molecular Formula	Molecular Weight	Retention Time	Reported Biological Activity
1	Ricinoleic acid	C_18_H_34_O_3_	298	51.979	Anti-inflammatory [27]
2	Gamabufotalin	C_24_H_34_O_5_	402	52.658	Anti-inflammatory [28]
3	Colchicine	C_22_H_25_NO_6_	399	53.938	Anti-inflammatory [29]
4	Beclomethasone	C_22_H_29_CIO_5_	408	53.938	Anti-inflammatory [30]
5	Prednisone	C_21_H_26_O_5_	358	56.624	Anti-inflammatory [31]
6	ß Carotene	C_4_0H_56_	536	57.838	Antioxidant and anti-inflammatory [32]
7	Levodopa	C_9_H_11_NO_4_	197	57.838	Anti-inflammatory [33]
8	Retinol	C_20_H_30_O	286	59.240	Anti-obesity [34]
9	Triaziquone	C_12_H_13_N_3_O_2_	231	61.136	Anti-inflammatory, Anti-cancer [35]
10	Retinyl acetate	C_22_H_32_O_2_	328	69.637	Anti-proliferation [34]
11	Vincamine	C_21_H_26_N_2_O_3_	354	71.044	Anti-aging, Anti-inflammatory [36]

**Table 2:** Effect of basil seed methanol extract (BSME) on triglyceride level, glycerol phosphate dehydrogenase and lactate dehydrogenase activity after 14 days in maturing adipocytes.

Groups	Glycerol Phosphate Dehydrogenase (Unit */mg Protein)	Triglyceride (mg/dL)	Lactate Dehydrogenase ^†^
Vehicle Control	105.98 ± 44.56	5.29 ± 1.74	0.18 ± 0.01
BSME (80 µg/dL)	54.6 ± 6.43 *	3.65 ± 1.05 *	0.11 ± 0.01 *
BSME (160 µg/dL)	25.7 ± 2.47 **	3.24 ± 1.09 **	0.10 ± 0.01 **
Tea Polyphenol (160 µg/dL)	88.7 ± 7.26 *	3.85 ± 1.16 *	0.13 ± 0.02 *

Values are means ± SD (*n* = 6). * 1 unit = 1 nmol/mg protein; ^†^ 1 mU/mg protein of LDH activity Å 1 nmole of NADH oxidized per minute per mg protein. * *p* ≤ 0.05 by comparison with control and ** *p* ≤ 0.001 by comparison with vehicle control and tea polyphenol.

## Data Availability

Not applicable.

## Supplementary Materials

The following supporting information can be downloaded, Figure S1: GC-MS spectral analysis data for basil seed hexane extract; Table S1: Basil seed hexane extract composition analysis using GC–MS shown 95–99% similarity in the database.

## References

[1] Rebollo-Hernanz M., Zhang Q., Aguilera Y., Martín-Cabrejas M.A., de Mejia E.G. (2019). Cocoa Shell Aqueous Phenolic Extract Preserves Mitochondrial Function and Insulin Sensitivity by Attenuating Inflammation between Macrophages and Adipocytes In Vitro. Mol. Nutr. Food Res..

[2] Kim S.P., Nam S.H., Friedman M. (2015). Mechanism of the antiadipogenic-antiobesity effects of a rice hull smoke extract in 3T3-L1 preadipocyte cells and in mice on a high-fat diet. Food Funct..

[3] Hammarstedt A., Gogg S., Hedjazifar S., Nerstedt A., Smith U. (2018). Impaired adipogenesis and dysfunctional adipose tissue in human hypertrophic obesity. Physiol. Rev..

[4] Choi S.S., Park J., Choi J.H. (2014). Revisiting PPARγ as a target for the treatment of metabolic disorders. BMB Rep..

[5] Meissburger B., Ukropec J., Roeder E., Beaton N., Geiger M., Teupser D., Civan B., Langhans W., Nawroth P.P., Gasperikova D. (2011). Adipogenesis and insulin sensitivity in obesity are regulated by retinoid-related orphan receptor gamma. EMBO Mol. Med..

[6] Neeland I.J., Turer A.T., Ayers C.R., Berry J.D., Rohatgi A., Das S.R., Khera A., Vega G.L., McGuire D.K., Grundy S.M. (2015). Body fat distribution and incident cardiovascular disease in obese adults. J. Am. Coll. Cardiol..

[7] Xiao J., Wang N.L., Sun B., Cai G.P. (2010). Estrogen receptor mediates the effects of pseudoprotodiocsin on adipogenesis in 3T3-L1 cells. Am. J. Physiol. Cell Physiol..

[8] Nicholson T., Church C., Baker D.J., Simon W.J. (2018). The role of adipokines in skeletal muscle inflammation and insulin sensitivity. J. Inflamm..

[9] Mancuso P. (2016). The role of adipokines in chronic inflammation. Immunotargets Ther..

[10] Yin X., Lanza I.R., Swain J.M., Sarr M.G., Nair K.S., Jensen M.D. (2014). Adipocyte mitochondrial function is reduced in human obesity independent of fat cell size. J. Clin. Endocrinol. Metab..

[11] Brand M.D., Orr A.L., Perevoshchikova I.V., Quinlan C.L. (2013). The role of mitochondrial function and cellular bioenergetics in ageing and disease. Br. J. Dermatol..

[12] Louet J.F., Chatelain F., Decaux J.F., Park E.A., Kohl C., Pineau T., Girard J., Pegorier J.P. (2001). Long-chain fatty acids regulate liver carnitine palmitoyltransferase I gene (L-CPT I) expression through a peroxisome-proliferator-activated receptor alpha (PPARalpha)-independent pathway. Biochem. J..

[13] Blaser H., Dostert C., Mak T.W., Brenner D. (2016). TNF and ROS crosstalk in inflammation. Trends Cell Biol..

[14] Zhang J., Wang X., Vikash V., Ye Q., Wu D., Liu Y., Dong W. (2016). ROS and ROS-mediated cellular signaling. Oxidative Med. Cell. Longev..

[15] Lumeng C.N., Deyoung S.M., Bodzin J.L., Saltiel A.R. (2007). Increased inflammatory properties of adipose tissue macrophages recruited during diet-induced obesity. Diabetes.

[16] Takeuchi H., Takahashi-Muto C., Nagase M., Kassai M., Tanaka-Yachi R., Kiyose C. (2020). Anti-inflammatory effects of extracts of sweet basil (*Ocimum basilicum* L.) on a co-culture of 3T3-L1 adipocytes and RAW264.7 macrophages. J. Oleo Sci..

[17] Noor Z.I., Ahmed D., Rehman H.M., Qamar M.T., Froeyen M., Ahmad S., Mirza M.U. (2019). In Vitro antidiabetic, anti-obesity and antioxidant analysis of *Ocimum basilicum* aerial biomass and in silico molecular docking simulations with alpha-amylase and lipase enzymes. Biology.

[18] Calderón Bravo H., Vera Céspedes N., Zura-Bravo L., Muñoz L.A. (2021). Basil seeds as a novel food, source of nutrients and functional ingredients with beneficial properties: A Review. Foods.

[19] Subash-Babu P., Al-Maiman S.A., Al-Harbi L.N., Alshatwi A.A. (2020). Beneficial fatty acid ratio of *Salvia hispanica* L. (Chia Seed) potentially inhibits adipocyte hypertrophy, and decreases adipokines expression and inflammation in macrophage. Foods.

[20] Mosmann T. (1983). Rapid colorimetric assay for cellular growth and survival: Application to proliferation and cytotoxicity assays. J. Immunol. Methods.

[21] Park H.S., Kim G.H., Shim S.M. (2014). Different effect of methanol extracts and bioaccessible fraction of *S. milax* china on triglyceride accumulation in adipocytes. J. Food Biochem..

[22] Kim M.S., Kim J.K., Kwon D.Y., Park R. (2004). Anti-adipogenic effects of Garcinia extract on the lipid droplet accumulation and the expression of transcription factor. Biofactors.

[23] Bradford M.M. (1967). A rapid and sensitive method for the quantitation of microgram quantities of protein utilizing the principle of protein dye binding. An. Biochem..

[24] Yuan J.S., Reed A., Chen F., Stewart C.N. (2006). Statistical analysis of real-time PCR data. BMC Bioinform..

[25] Kim H.Y. (2014). Analysis of variance (ANOVA) comparing means of more than two groups. Restor. Dent. Endod..

[26] Filip S. (2017). Basil (Ocimum basilicum L.) a source of valuable phytonutrients. Int. J. Clin. Nutr. Diet..

[27] Vieira C., Fetzer S., Sauer S.K., Evangelista S., Averbeck B., Kress M., Reeh P.W., Cirillo A.L., Maggi C.A., Manzini S. (2001). Pro-and anti-inflammatory actions of ricinoleic acid: Similarities and differences with capsaicin. Naunyn-Schmiedeberg’s Arch. Pharmacol..

[28] Yu Z., Guo W., Ma X., Zhang B., Dong P., Huang L., Wang X., Wang C., Huo X., Yu W. (2014). Gamabufotalin, a bufadienolide compound from toad venom, suppresses COX-2 expression through targeting IKKβ/NF-κB signaling pathway in lung cancer cells. Mol. Cancer.

[29] Ben-Chetrit E., Bergmann S., Sood R. (2006). Mechanism of the anti-inflammatory effect of colchicine in rheumatic diseases: A possible new outlook through microarray analysis. Rheumatology.

[30] Robroeks C.M., Van de Kant K.D., Van Vliet D., Kester A.D., Hendriks H.J., Damoiseaux J.G., Wodzig W.K.W.H., Rijkers G.T., Dompeling E., Jobsis Q. (2008). Comparison of the anti-inflammatory effects of extra-fine hydrofluoroalkane-beclomethasone Vs fluticasone dry powder inhaler on exhaled inflammatory markers in childhood asthma. Ann. Allergy Asthma Immunol..

[31] Yan S.X., Deng X.M., Wang Q.T., Sun X.J., Wei W. (2015). Prednisone treatment inhibits the differentiation of B lymphocytes into plasma cells in MRL/MpSlac-lpr mice. Acta Pharmacol. Sin..

[32] Ciccone M.M., Cortese F., Gesualdo M., Carbonara S., Zito A., Ricci G., De Pascalis F., Scicchitano P., Riccioni G. (2013). Dietary intake of carotenoids and their antioxidant and anti-inflammatory effects in cardiovascular care. Mediat. Inflamm..

[33] Maccarrone M., Gubellini P., Bari M., Picconi B., Battista N., Centonze D., Bernardi G., Finazzi-Agrò A., Calabresi P. (2003). Levodopa treatment reverses endocannabinoid system abnormalities in experimental parkinsonism. J. Neurochem..

[34] Botella-Carretero J.I., Balsa J.A., Vázquez C., Peromingo R., Díaz-Enriquez M., Escobar-Morreale H.F. (2010). Retinol and α-tocopherol in morbid obesity and nonalcoholic fatty liver disease. Obes. Surg..

[35] Huang C.H., Kuo H.S., Liu J.W., Lin Y.L. (2009). Synthesis and antitumor evaluation of novel bis-triaziquone derivatives. Molecules.

[36] Fayed A.H.A. (2010). Brain trace element concentration of rats treated with the plant alkaloid, vincamine. Biol. Trace Elem. Res..

[37] Baliga M.S., Jimmy R., Thilakchand K.R., Sunitha V., Bhat N.R., Saldanha E., Rao S., Arora R., Palatty P.L. (2013). Ocimum sanctum L (Holy Basil or Tulsi) and its phytochemicals in the prevention and treatment of cancer. Nutr. Cancer.

[38] Bucktowar K., Bucktowar M., Bholoa L.D. (2016). A review on sweet basil seeds: Ocimum basilicum. World J. Pharm. Pharm. Sci..

[39] Yang Y., Wang J., Zhang Y., Li J., Sun W. (2018). Black sesame seeds ethanol extract ameliorates hepatic lipid accumulation, oxidative stress, and insulin resistance in fructose-induced nonalcoholic fatty liver disease. J. Agric. Food Chem..

[40] Wu L., Zhang L., Li B., Jiang H., Duan Y., Xie Z., Shuai L., Li J., Li J. (2018). AMP-activated protein kinase (AMPK) regulates energy metabolism through modulating thermogenesis in adipose tissue. Front. Physiol..

[41] Laura L.G., Stuart A.M., Jeremy W.T. (2013). Hormonal Regulation of Lipogenesis. Vitam. Horm..

[42] Luo J., Qi J., Wang W., Luo Z., Liu L., Zhang G., Zhou Q., Liu J., Peng X. (2019). Antiobesity effect of flaxseed polysaccharide via inducing satiety due to leptin resistance removal and promoting lipid metabolism through the AMP-activated protein kinase (AMPK) signaling pathway. J. Agric. Food Chem..

[43] Berndt J., Kralisch S., Klöting N., Ruschke K., Kern M., Fasshauer M., Schön M.R., Stumvoll M., Blüher M. (2008). Adipose triglyceride lipase gene expression in human visceral obesity. Exp. Clin. Endocrinol. Diabetes.

[44] Gao X., Li K., Hui X., Kong X., Sweeney G., Wang Y., Xu A., Teng M., Liu P., Wu D. (2011). Carnitine palmitoyltransferase 1A prevents fatty acid-induced adipocyte dysfunction through suppression of c-Jun N-terminal kinase. Biochem. J..

[45] Serra D., Mera P., Malandrino M.I., Mir J.F., Herrero L. (2013). Mitochondrial fatty acid oxidation in obesity. Antioxid. Redox Signal..

[46] Chani E.M.M., Pacheco S.O.S., Martínez G.A., Freitas M.R., Ivona J.G., Ivona J.A., Craig W.J., Pacheco F.J. (2018). Long-Term dietary intake of chia seed is associated with increased bone mineral content and improved hepatic and intestinal morphology in Sprague-Dawley Rats. Nutrients.

[47] Coppack S.W. (2001). Pro-inflammatory cytokines and adipose tissue. Proc. Nutr. Soc..

[48] Ouchi N., Parker J.L., Lugus J.J., Walsh K. (2011). Adipokines in inflammation and metabolic disease. Nat. Rev. Immunol..

[49] Fève B., Bastard J.P. (2009). The role of interleukins in insulin resistance and type 2 diabetes mellitus. Nat. Rev. Endocrinol..

[50] Vozarova B., Weyer C., Hanson K., Tataranni P.A., Bogardus C., Pratley R.E. (2001). Circulating interleukin-6 in relation to adiposity P.A., insulin action, and insulin secretion. Obes. Res..

[51] Makki K., Froguel P., Wolowczuk I. (2013). Adipose tissue in obesity-related inflammation and insulin resistance: Cells, cytokines, and chemokines. ISRN Inflamm..

[52] Corrêa L.H., Corrêa R., Farinasso C.M., de Sant’Ana D.L.P., Magalhães K.G. (2017). Adipocytes and Macrophages Interplay in the Orchestration of Tumor Microenvironment: New Implications in Cancer Progression. Front. Immunol..

[53] Plomgaard P., Bouzakri K., Krogh-Madsen R., Mittendorfer B., Zierath J.R., Pedersen B.K. (2005). Tumor necrosis factor-alpha induces skeletal muscle insulin resistance in healthy human subjects via inhibition of Akt substrate 160 phosphorylation. Diabetes.

[54] Weisberg S.P., McCann D., Desai M., Rosenbaum M., Leibel R.L., Ferrante A.W. (2003). Obesity is associated with macrophage accumulation in adipose tissue. J. Clin. Investig..

[55] Lumeng C.N., Deyoung S.M., Saltiel A.R. (2007). Macrophages block insulin action in adipocytes by altering expression of signaling and glucose transport proteins. Am. J. Physiol. -Endocrinol. Metab..

