# Supplementary material for: Ocimum basilicum L. Methanol Extract Enhances Mitochondrial Efficiency and Decreases Adipokine Levels in Maturing Adipocytes Which Regulate Macrophage Systemic Inflammation
Source: Molecules. 2022 Feb 18;27(4):1388. doi: 10.3390/molecules27041388 (PMC8876186; doi:10.3390/molecules27041388)
Supplement: Supplementary file 1 [file molecules-27-01388-s001.zip › molecules-1549859-SI.pdf]

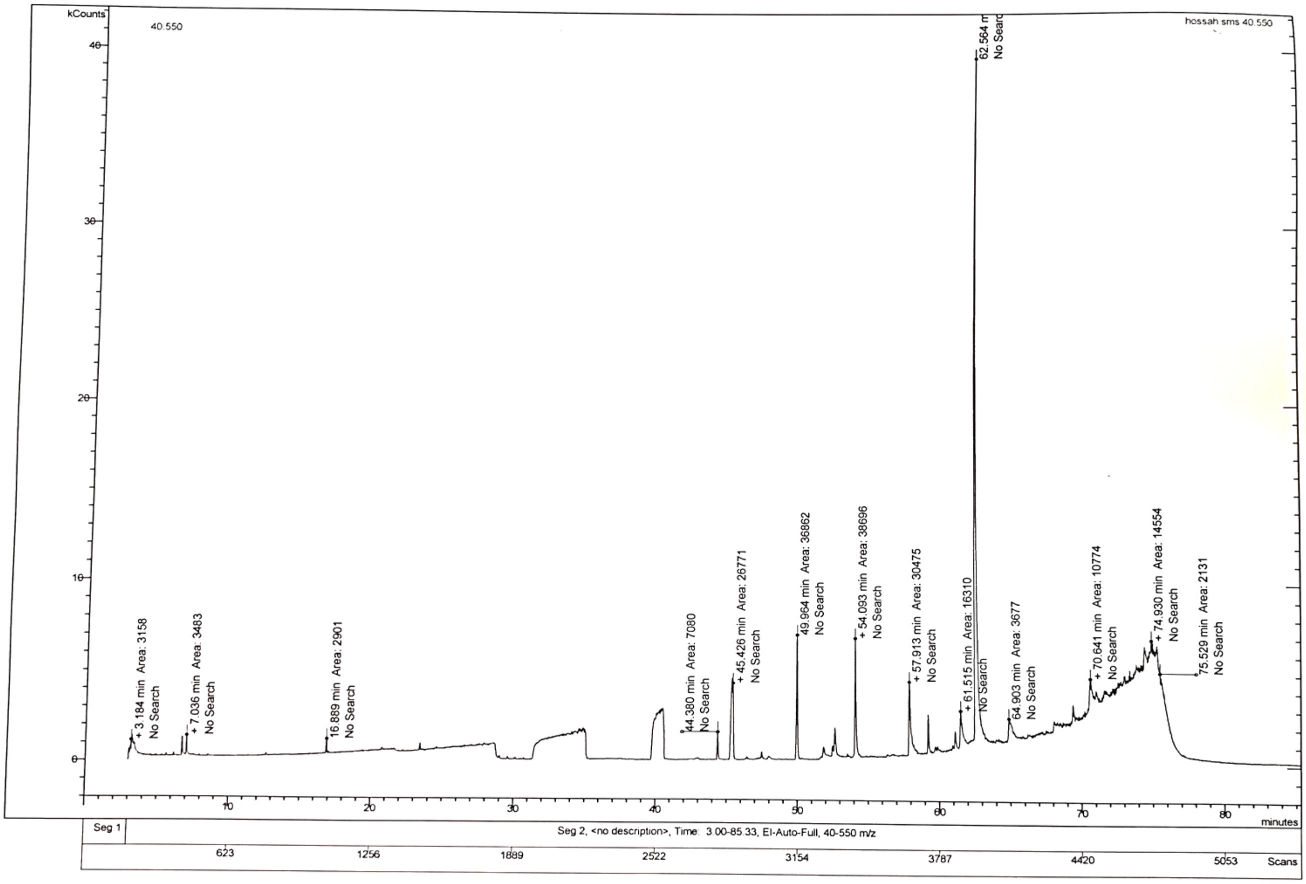

Figure S1. GC-MS spectral analysis data for Basil seed hexane extract.

Table S1. Basil Seed Hexane extract composition analysis using GC–MS shown 99–95%similarity in the database.

Peak No	Compounds	Molecular formula	Molecular weight	Retention time
1	(2S)-2,3-Dihydroxybutanedioic acid	C4H6O6	150	3.231
2	Octodrine	C8H19N	129	7.024
3	4a,7-Methano-4aH-naphthoxirene, octahydro-4,4,8,8-tetramethyl-	$C_{15}H_{24}O$	220	44.399
4	Colchicine	$C_{22}H_{25}NO_6$	399	45.411
5	Gamabufotalin	C24H34O5	402	49.979
6	Ricinoleic acid	$C_{18}H_{34}O_3$	298	52.649
7	Retinol	C20H30O	286	59.250
8	ß Carotene	$C_{40}H_{56}$	536	64.912
9	Retinyl acetate	C22H32O2	328	73.714

GC-MS chromatogram of basil seed hexane extract have been presented in S-1 and T-1. Spectral data were compared with NIST-11 library and identified phytochemicals, we found 99%-95% similarity as per the peak values and retention time. Basil seed hexane extract also shown the availability of dihydroxybutanedioic acid, octodriene, colchicine, gamabufotalin, retinol, β carotene and retinyl acetate.
